# Prediction of survival prognosis after surgery in patients with symptomatic metastatic spinal cord compression from non-small cell lung cancer

**DOI:** 10.1186/s12885-015-1852-2

**Published:** 2015-11-05

**Authors:** Mingxing Lei, Yaosheng Liu, Chuanghao Tang, Shaoxing Yang, Shubin Liu, Shiguo Zhou

**Affiliations:** Department of Orthopedic Surgery, Affiliated Hospital of Academy of Military Medical Sciences, No. 8, Fengtaidongda Rd, Beijing, 100071 People’s Republic of China; Department of Pulmonary Neoplasms Internal Medicine, Affiliated Hospital of Academy of Military Medical Sciences, No. 8, Fengtaidongda Rd, Beijing, China; Statistics Room, Capital Medical University affiliated Beijing Friendship Hospital, No. 95, Xuanwu District Yongan Rd, Beijing, China

**Keywords:** Metastatic spinal cord compression, Non-small cell lung cancer, Surgery, Score, Survival, Prediction

## Abstract

**Background:**

The aim of this study was to develop a scoring system for prediction of survival prognosis after surgery in patients with symptomatic metastatic spinal cord compression (MSCC) from non-small cell lung cancer (NSCLC).

**Methods:**

We retrospectively analyzed nine preoperative characteristics for survival in a series of 64 patients with NSCLC who were operated with posterior decompression and spine stabilization for MSCC. Characteristics significantly associated with survival on multivariate analysis were included in the scoring system. The scoring point for each significant characteristic was derived from the hazard ratios on Cox proportional hazards model. The total score for each patient was obtained by adding the scoring points of all significant characteristics.

**Results:**

Eastern Cooperative Oncology Group (ECOG) performance status, number of involved vertebrae, visceral metastases, and time developing motor deficits had significant impact on survival on multivariate analysis and were included in the scoring system. According to the prognostic scores, which ranged from 4 to 10 points, three prognostic groups were designed: 4–5 points (*n* = 22), 6–7 points (*n* = 23), and 8–10 points (*n* = 19). The corresponding 6-month survival rates were 95, 47 and 11 %, respectively (*P* < 0.0001). In addition, the functional outcome was worse in the group of patients with 8–10 points compared with other two prognostic groups.

**Conclusions:**

The new scoring system will enable physicians to identify patient with MSCC from NSCLC who may be a candidate for decompression and spine stabilization, more radical surgery, or supportive care alone. Patients with scores of 4–5, who have the most favorable survival prognosis and functional outcome, can be treated with more radical surgery in order to realize better local control of disease and prevent the occurrence of local disease. Patients with scores of 6–7 points should be surgical candidates, because survival prognosis and functional outcome are acceptable after surgery, while patients with scores of 8–10 points, who have the shortest survival time and poorest functional outcome after surgery, appear to be best treated with radiotherapy or best supportive care.

## Background

Metastatic spinal cord compression (MSCC) is a severe complication of cancer that occurs in 28 % of patients with lung cancer and can become symptomatic, which involves intractable pain, disability, and incontinence [1–3], negatively impacting the patient's quality of remaining life. The optimal treatments for patients with MSCC are analgesics, corticosteroids, chemotherapy, radiotherapy and surgery, and most often these treatments are combined to give the maximum palliative effect with a minimum of operative morbidity and mortality [1, 4, 5], positively improving the patient's quality of remaining life. Recently, an increasing number of studies supported the use of decompressive surgery as an effective treatment for MSCC due to the evolvement of surgical techniques [1, 2, 6], while only a few studies specifically addressed surgical treatment of MSCC in lung cancer [7, 8], which was often associated with high morbidity and mortality [8]. A major problem in selection patients for surgery is to avoid operating on those who are likely to die very soon after surgery, so life expectancy is the most important selection criteria for surgery. While for patients with very short survival time radiotherapy or best supportive care alone are recommended, for patients with more favorable prognosis can be treated with decompressive surgery, or even more radical surgery such as excisional procedures [4, 9, 10].

Some scoring systems were designed to estimate the survival time of each patient and select the optimal treatment strategy among supportive care, palliative radiotherapy, palliative surgery, and excisional surgery [9–15]. However, some old and commonly-used scoring systems have underestimated the life expectancy of lung cancer patients with spinal metastases because of the increased survival time for this patient group in recent years [16–19]. Notably, it is critical to regard patients with MSCC from a particular primary tumor type as a separate group of patients for individual treatment, because primary tumors vary with respect to their biological behavior. Therefore, our present study is designed to develop a new survival score particularly for patients with MSCC from non-small cell lung cancer (NSCLC) after surgery.

## Methods

### Patients

Sixty-four patients with NSCLC operated with decompression and spine stabilization for MSCC were retrospectively analyzed in the study at the Affiliated Hospital of Academy of Military Medical Sciences, Beijing, between May 2005 and May 2015. The diagnosis of bone metastasis in NSCLC patients was confirmed histologically, adequate diagnostic imaging including spinal CT or MRI, as well as bone scan. Patients with an estimated survival less than 3 months or health too poor to undergo surgery were excluded. Of the total series of 64 patients, six patients were treated with radical resection of primary lung cancer, while others weren’t. The data were collected from patients, their family members, treating surgeons, and patients’ files. The Medical Research Ethics Board of the Affiliated Hospital of Academy of Military Medical Sciences approved this retrospective study and required neither patient approval nor informed consent for review of patients’ images and medical records. The data were retrospective in nature and anonymized by the Medical Research Ethics Board.

### Survival analysis

We retrospectively analyzed nine preoperative characteristics for survival, including age (≤57 years vs. ≥58 years; median age: 57 years), gender (female vs. male), preoperative ambulatory status (ambulatory vs. nonambulatory), other bone metastases (no vs. yes), Eastern Cooperative Oncology Group (ECOG) performance status (1–2 vs. 3–4), number of involved vertebrae (1–2 vs. ≥3, conformed to previous studies), visceral metastases (no vs. yes), interval from cancer diagnosis to surgery (≤80 days vs. >80 days; median time: 80 days), and the time developing motor deficits before surgery (≤14 days vs. >14 days, conformed to previous studies).

The postoperative survival was defined as the time between the date of surgery and death or the latest follow-up. For the present study, we included all 64 patients with NSCLC who had decompressive surgery and spine stabilization due to spinal cord compression. None of the patients were excluded for any reason. 5 patients were still alive by the end of the study period, with a mean follow-up of 9.7 months in those patients. In patients who had surgery for more than one metastasis, all sites were included in the analysis. However, only the first surgical procedure was accounted for in the survival analysis.

### Surgery and functional evaluation

The indication for surgery was neurological deficit due to spinal cord compression. All patients were operated with posterior decompression and stabilization in our department. Local radiotherapy, systemic chemotherapy, and targeted therapy with gefitinib were performed after the wound healed, about 3–4 weeks after the surgery. Postoperative functional outcome was analyzed according to the scoring system. Neurological function was graded based on Frankel et al. [20] preoperatively and 4 weeks postoperatively (Patients with Frankel D and E have the ability to walk). Time developing motor deficits was defined as the time between deterioration of motor function to disability or surgery. Deterioration of motor function was defined as a change of at least one Frankel grade.

### Statistical analysis

The univariate analysis of survival was performed using the Kaplan-Meier method and the log-rank test. The significant prognostic factors (*P* < 0.05) were additionally evaluated in a multivariate analysis performed with the Cox proportion hazards model (multiple Cox regression, selection = stepwise). The prognostic factors that were significant in the multivariate analysis were included in the scoring system. The prognostic factors that were excluded by Cox proportion hazards model (multiple Cox regression, selection = stepwise) were not included in the scoring system. The scoring point for each significant factors was derived from the hazard ratios on Cox proportional hazards model (simple Cox regression). The total prognostic score for each patient was determined by adding the scoring points of every significant factor. Neurological outcome in risk groups was compared with Chi-square test and Fisher exact test. A *P* value of *0.05* or less was considered statistically significant. Statistical analysis was performed using SAS 9.2 software.

## Results

### Patient characteristics and survival

A total of 64 patients were included in the study, 34 % (22/64) of patients were female, and 66 % (42/64) were male. The overall median age was 57 years old. The median time interval from diagnosis to surgery was 80 days, and the median time developing motor deficits was 14 days.

For all patients, the overall median survival time was 6.3 months (95 % confidence interval, 4.5–7.4 months), 6-month and 12-month survival rates were 52.6 and 23 %, respectively. At the latest follow-up, 5 patients were still alive, with a mean follow-up of 9.7 months.

### Scoring system

**Fig. 1:**
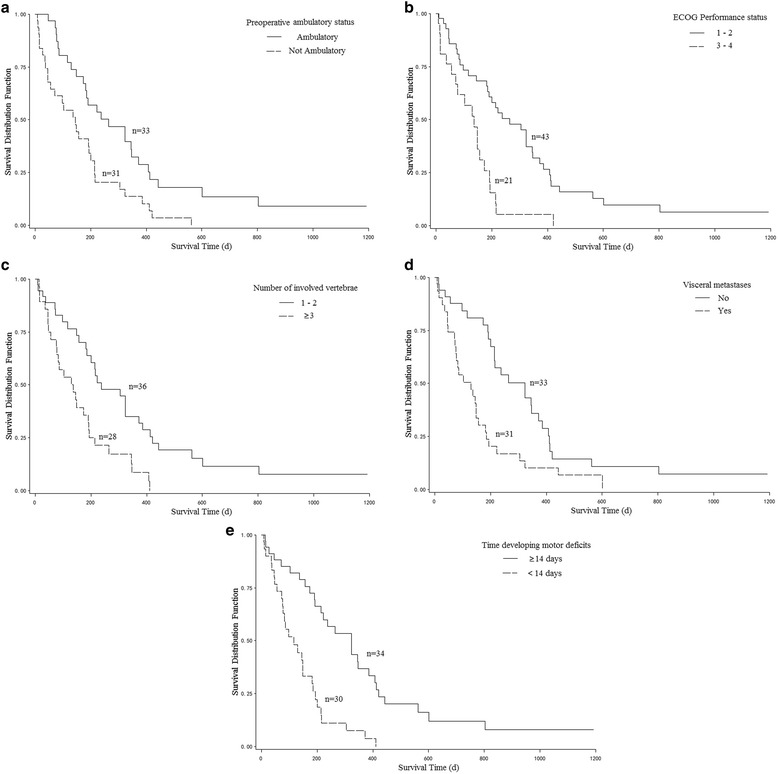
Kaplan-Meier survival curves for preoperative factors: (**a**) Preoperative ambulatory status, (**b**) ECOG performance status, (**c**) Number of involved vertebrae, (**d**) Visceral metastases, and (**e**) Time developing motor deficits

**Fig. 2:**
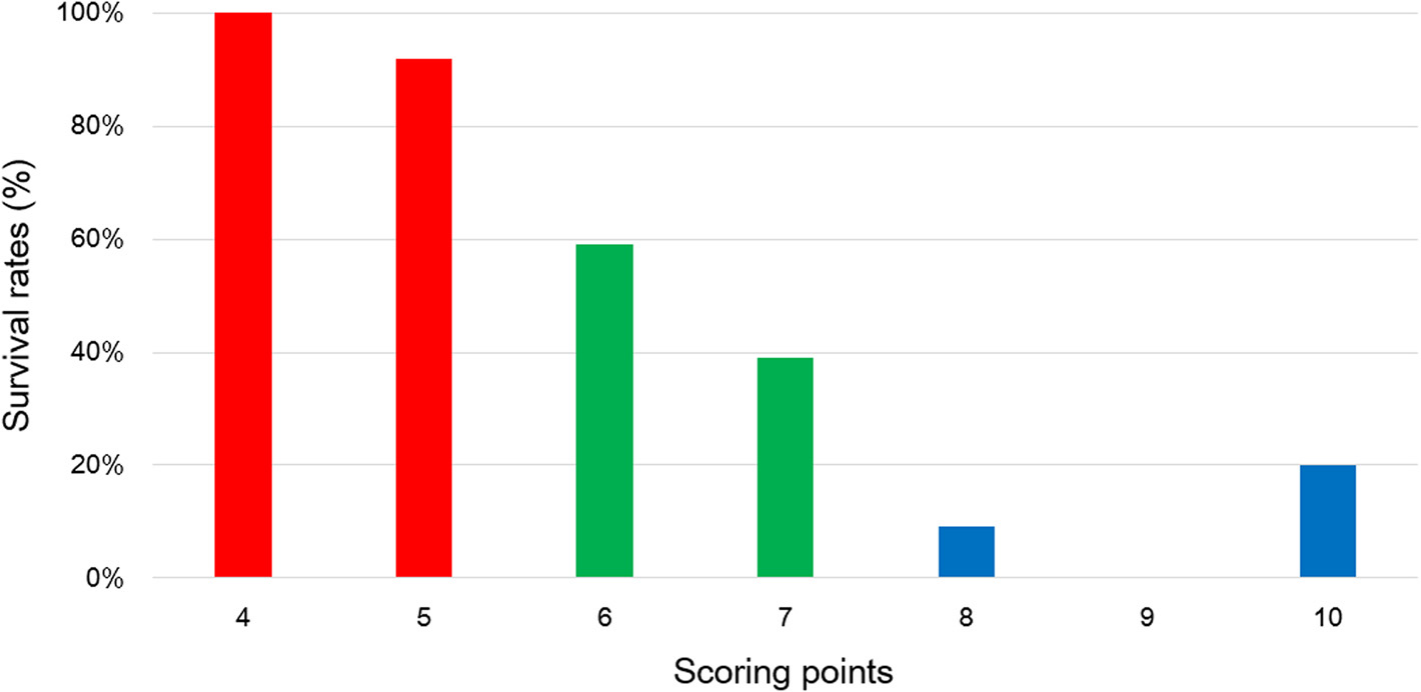
The total scores and corresponding 6-month survival rates (%)

**Fig. 3:**
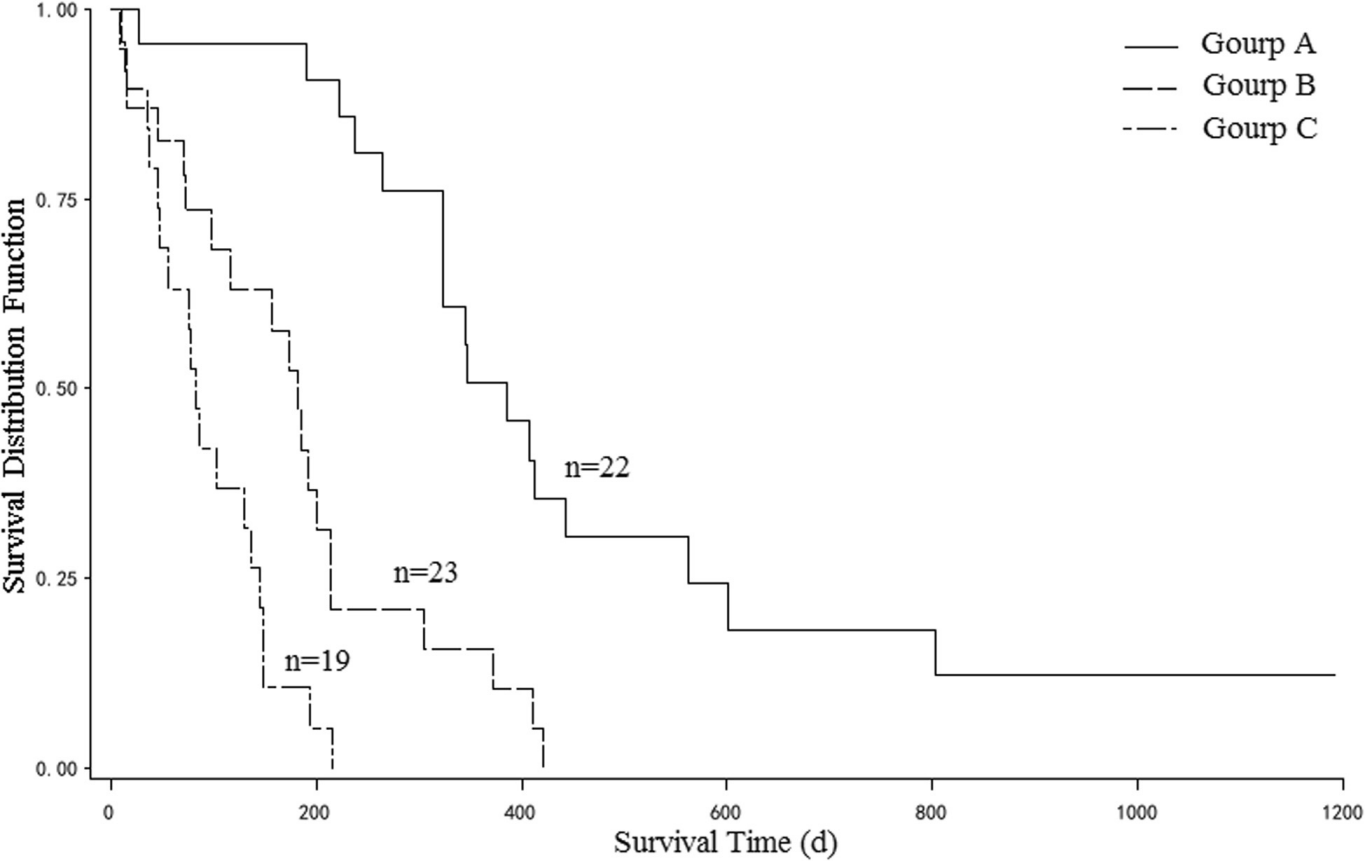
Kaplan-Meier survival curves for three prognostic groups based on the new scoring system (*P* < 0.001, log-rank test)

On the univariate analysis, survival was significantly associated with preoperative ambulatory status (*P* = 0.003), ECOG performance status (*P* < 0.001), number of involved vertebrae (*P* = 0.001), visceral metastases (*P* = 0.002), and time developing motor deficits (*P* < 0.001, Fig. 1, Table 1). On Cox proportional hazards model (multiple Cox regression, selection = stepwise), four of above five factors, ECOG performance status (*P* = 0.017), number of involved vertebrae (*P* = 0.021), visceral metastases (*P* = 0.022), and time developing motor deficits (*P* = 0.002), maintained significant impact on survival and were included in the scoring system (Table 2). The scoring points for each of the four significant factors obtained from the hazard ratios on Cox proportional hazards model (simple Cox regression) were seen in Table 3. The prognostic score for each patient was calculated by adding the scoring points of the four significant characteristics. The addition resulted in prognostic scores of 4, 5, 6, 7, 8, 9, 10 points. The 6-month survival rates of the prognostic scores were shown in Fig. 2. Taking into account the 6-month survival rates of the prognostic scores, the following three survival groups were formed: 4–5 points (group A, *n* = 22), 6–7 points (group B, *n* = 23), and 8–10 points (group C, *n* = 19). The corresponding median survival times were 12.8 months (95 % confidence interval, 8.8–18.7 months), 6.4 months (95 % confidence interval, 3.8–7.4 months) and 2.7 months (95 % confidence interval, 1.5–4.5 months), respectively, and 6-month survival rates were 95, 47 and 11 %, respectively (*P* < 0.001, Fig. 3).

**Table 1:** Univariate analysis of preoperative factors for postoperative survival in patients with MSCC from NSCLC

Factors	Patients (n)	Survival		MOS (mo)	*P*
		6 mo (%)	12 mo (%)		
Age					
≤ 57 years	34	61	27	7.1	0.16
≥ 58 years	30	42	18	4.8	
Gender					
Female	22	55	23	6.3	0.90
Male	42	52	24	6.2	
Preoperative ambulatory status					
Ambulatory	33	64	32	8.8	0.003
Nonambulatory	31	41	14	4.8	
Other bone metastases					
No	16	69	21	7.9	0.58
Yes	48	47	23	4.9	
ECOG performance status					
1–2	43	66	29	8.8	<0.001
3–4	21	26	5	4.5	
Number of involved vertebrae					
1–2	36	67	35	7.9	0.001
≥ 3	28	36	9	4.4	
Visceral metastases					
No	33	78	36	10.8	0.002
Yes	31	27	10	4.3	
Interval from cancer diagnosis to surgery					
≤ 80 days	32	59	24	7.1	0.73
> 80 days	32	47	22	5.5	
Time developing motor deficits					
≤ 14 days	30	30	7	3.9	<0.001
> 14 days	34	72	37	10.8	

*MSCC* indicates metastatic spinal cord compression; *NSCLC*, non-small cell lung cancer; *MOS*, median overall survival; *MO*, months; *ECOG*, Eastern Cooperative Oncology Group

**Table 2:** The Cox proportional hazards model analysis of preoperative factors for postoperative survival in patients with MSCC from NSCLC

Factors	Simple cox regression		Multiple cox regression	
	HR (95 % CI)	*P*	HR (95 % CI)	*P*
Preoperative ambulatory status	2.24 (1.30–3.86)	0.004	Excluded^a^	
ECOG performance status	2.78 (1.54–5.02)	<0.001	2.18 (1.15–4.16)	0.017
Number of involved vertebrae	2.46 (1.39–4.35)	0.002	2.05 (1.11–3.76)	0.021
Visceral metastases	2.29 (1.33–3.94)	0.003	2.00 (1.10–3.62)	0.022
Time developing motor deficits	3.44 (1.90–6.22)	<0.001	2.70 (1.45–5.03)	0.002

*MSCC* indicates metastatic spinal cord compression; *NSCLC*, non-small cell lung cancer; *ECOG*, Eastern Cooperative Oncology Group; *HR*, hazard ratio; *CI*, confidence interval

^a^Selection = stepwise, preoperative ambulatory status was excluded in the model

**Table 3:** Hazard ratio and corresponding scores of each significant factors in the scoring system

Factors	Patients (*n*)	HR	Scoring points
ECOG performance status			
1–2	43	1	1
2–4	21	2.78	3
Number of involved vertebrae			
1–2	36	1	1
≥ 3	28	2.46	2
Visceral metastases			
No	33	1	1
Yes	31	2.29	2
Time developing motor deficits			
< 14 days	30	3.44	3
≥ 14 days	34	1	1

*HR* indicates hazard ratio, *ECOG* Eastern Cooperative Oncology Group

### Functional outcome

The functional outcome was worse in the group of patient with 8–10 points (group C) compared with the other two prognostic groups (Table 4). In detail, 86 % (19/22) patients were ambulatory 4 weeks after surgery in group A, 74 % (17/23) patients in group B, and only 42 % (8/19) patients in group C.

**Table 4:** Neurological recovery of the patients in 3 prognostic groups 4 weeks after surgery

Groups	Scores	Patients(*n*)	Neurological status weeks postoperation		*P* values
			Ambulatory	Nonambulatory	
A	4–5	22	19	3	*P*1 = 0.502
B	6–7	23	17	6	*P*2 = 0.037
C	8–10	19	8	11^a^	*P*3 = 0.003

*P*1 Group A compared with group B, Continuity Adjusted Chi-square test;

*P*2 Group B compared with group C, Chi-square test;

*P*3 Group C compared with group A, Chi-square test

^a^2 patients died within 4 weeks, none of both realized ambulatory status

In the entire cohort of 64 patients, 68.8 % (44 of 64) of the patients were able to walk 4 weeks after decompression, 51.6 % (16/31) of nonambulatory patients before operation regained the ability to walk, and 84.8 % (28/33) of ambulatory patients maintained their neurological status, whereas 15.2 % (5/33) of ambulatory patients before surgery lost their ability to walk for disease progression. Six patients died within 4 weeks after surgery and none of them achieved ambulation.

## Discussion

Individually treatment needs to be planned for each patient with MSCC to give the maximum palliative effect: reduction in pain, recovery of function, and improvement in the patient’s quality of remaining life. Selection of the optimal treatment for the individual patient with MSCC should take into account patient’s estimated survival time, as well as functional outcome after therapies. Only those who survive long enough, more than 3 months, can benefit from surgery [21, 22]. In contrast, patients with very short survival time and poor functional outcome appear to be best treated with radiotherapy or even best supportive care alone, which means less discomfort for these debilitated and enervated patient [4, 10]. Remarkably, it is also critical to regard patients with MSCC from a particular primary tumor type as a separate group of patients for optimal treatment, because primary tumors vary with respect to their biological behavior. Crnalic et al. [23] presented a score specifically for predicting survival of patients with prostate cancer after surgery for MSCC. However, who may benefit from surgery, and what kind of patients are appropriate for supportive care, remains nuclear in NSCLC patients with MSCC.

Several scoring systems have been proposed for predicting survival in patient with spinal metastasis on the basis of retrospective data from various primary tumors treated with surgery or radiotherapy alone. However, these scores comprised relatively small number of patient with lung cancer (Tokuhashi 6 [9], revised Tokuhashi 26 [10], Tomita 10 [11], Van der Linden 68 [12], Sioutos 45 [13], Bauer 6 [14], Bartels 28 [15], more details were seen in Table 5), making it difficult to draw conclusions on this specific tumor type.

**Table 5:** Commonly-used and our scoring systems for patient with spinal metastases^a^

Scoring systems	MOS (m)	Suggestions	No. of LC (Total)	Spinal metastasis	Treatments	Parameters
Tokuhashi [9]						
Group A	3	Palliative surgery	6 (64)	In general	All surgery^c^	PS; Extraspinal bone metastases; Metastases in the vertebral body; Metastases to major organs; primary tumor site; Spinal cord palsy
Group B	6	-				
Group C	22	Excisional surgery				
Revised Tokuhashi [10]						
Group A	4.9	Conservation therapy	26 (246)	In general	164 patients was treated with surgery	PS; Extraspinal bone metastases; Metastases in the vertebral body; Metastases to major organs; Primary tumor site; Spinal cord palsy
Group B	9.5	Palliative surgery				
Group C	19	Excisional surgery				
Tomita [11]						
Group A	6	Supportive care	10 (67)	In general	58 patients was treated with surgery	No. of extraspinal bone metastases; Metastases to major internal organs; Primary tumor site; Spinal cord palsy
Group B	15	Palliative surgery				
Group C	24	Intralesional/marginal				
Group D	50	Excisional surgery				
Van der Linden [12]						
Group A	4.8	Radiotherapy	68 (324)	No MSCC	Radiotherapy alone	KPS; Primary tumor; Visceral metastases
Group B	13.1	Radiotherapy				
Group C	18.3	Surgery				
Sioutos [13]						
3^b^	1.5	No surgery	45 (109)	MSCC	All surgery^d^	Preoperative neurological status; Anatomic site of primary carcinoma; No. of vertebral bodies involved
2^b^	6.0	No surgery				
1^b^	11.2	Radical surgery				
0^b^	18.0	Radical surgery				
Bauer [14]						
Group A	-	No surgery	6 (88)	In general	All surgery^e^	Visceral metastases; No. of skeletal metastases; Primary cancer type.
Group B	-	Dorsal surgery				
Group C	-	Ventral-dorsal surgery				
Bartels [15]						
Not reported			28 (219)	In general	Radiotherapy alone	Sex; Location of the primary lesion; Curative treatment of the primary tumor; Location of the spinal metastasis; KPS
Ours						
Group A	12.8	More radical surgery	64 (64)	MSCC	All surgery^e^	ECOG performance status; No. of involved vertebrae; Visceral metastases; Time developing motor deficits.
Group B	6.4	Depressive surgery				
Group C	2.7	Supportive care				

*MOS* indicates mean overall survival; *LC*, lung cancer; *PS*, performance status; *KPS*, karnofsky performance status; *MSCC*, metastatic spinal cord compression, *ECOG*, Eastern Cooperative Oncology Group

^a^Functional outcome are not considered in all of their original studies

^b^No. of negative prognostic factors

^c^Excisional or palliative procedure

^d^Anterior or posterior approach

^e^Posterior approach

Although the revised Tokuhashi was found to be useful to predict survival for patients with spinal metastases from breast cancer alone [4] or solid cancers [24, 25], which seems to be a suboptimal tool for the prediction of an individual prognosis in the group of patients with lung cancer (Hessler et al. [16]). In their study, 67 patients with spinal metastasis from lung cancer, all of them underwent surgical treatment. Hessler et al. [16] concluded that the Tokuhashi scoring system underestimated the life expectancy of lung cancer patients due to the increased survival time for this patient group. In 2013, Morgen et al. [17] also found a statistically significant increase in survival over the years for lung cancer patients with MSCC (*n* = 2321, 499 patients with lung cancer, 103 lung cancer patients received surgical treatment). For patients with lung cancer who underwent surgery for MSCC, survival increased from 9 % in year 2005 up to 30 % in year 2010 (*P* = 0.047). More recent studies have reported improvements among patients with advanced lung cancer because of the new treatment options [18, 19]. Therefore, with the increasing survival time of patients with lung cancer during recent years, the Tokuhashi scoring system and other scores may no longer be suitable for patients with lung cancer.

Furthermore, these scores were designed for patients with spinal metastasis in general, not particularly for patients with motor impairment due to MSCC. Rades et al. [26] developed and validated a scoring system for survival of patients (*n* = 356, all patients with lung cancer) with MSCC from NSCLC who had been treated with radiotherapy alone. Aside from the Rades score, the above mentioned scoring systems included relatively small number of patients with spinal metastasis from various primary tumors. In fact, participants in Rades score received radiotherapy alone, and the functional outcome was not considered either. Moreover, patients who had prior surgery to the involved parts of the spinal cord were excluded in their study.

In our study, a score was developed based on the data derived from 64 patients with NSCLC who underwent decompressive surgery and spine stabilization for MSCC. The indication for surgery was neurological deficits. Functional outcome was also considered according to the scoring system. The patient’s individual situation, therefore, is taken more into account in the present scoring system. Patients with scores of 4–5 survived more than 1 year in median time, and 86 % patients were ambulatory 4 weeks after surgery. More radical surgery, such as widely excision of vertebra metastasis, can be considered in order to realize better local control of disease and prevent the occurrence of local disease in those patients. Patients with scores of 6–7 points should be surgical candidates, because survival prognosis and functional outcome were favorable after surgery. Patients with scores of 8–10 points, who survived 2.7 months in median time and had the worst functional outcome after surgery compared with other two prognostic groups, appeared to be best treated with radiotherapy or best supportive care alone. Functional outcome was acceptable in the entire cohort of 64 patients, 68.8 % (44 of 64) patients were able to walk 4 weeks after decompression; 51.6 % (16/31) of nonambulatory patients before operation regained the ability to walk. 74–84 % patients were able to walk after surgery [6, 7, 27] and 22–68 % of nonambulatory patients became ambulatory again in other studies [7,28].

However, patients with asymptomatic MSCC were not included in our study, so this scoring system doesn’t pertain to those patients. Besides, our score was based on retrospective data, and the statistical analysis didn’t include a relatively larger number of patients, and data on systemic treatment following treatment was not available in most patients. Despite good predictive value in our scoring system, the score still warrants a prospective study to be confirmed.

## Conclusion

We present a new score for predicting survival of patients with NSCLC operated with posterior decompression and spine stabilization for MSCC. Functional outcome after surgery was also considered in our study. The scoring system can help select the individual treatment for patients with MSCC from NSCLC. Patients with scores of 4–5, who have the most favorable survival prognosis and functional outcome, can be treated with more radical surgery in order to realize better local control of disease and prevent the occurrence of local disease. Patients with scores of 6–7 points should be surgical candidates, because survival prognosis and functional outcome are acceptable after surgery, while patients with scores of 8–10 points, who have the shortest survival time and poorest functional outcome after surgery, appear to be best treated with radiotherapy or best supportive care. Still, a prospective study is needed.

## Abbreviations

ECOG: Eastern Cooperative Oncology Group
MSCC: metastatic spinal cord compression
NSCLC: non-small cell lung cancer
